# Odor discrimination is immune to the effects of verbal labels

**DOI:** 10.1038/s41598-023-28134-w

**Published:** 2023-01-31

**Authors:** Sarah Cormiea, Jason Fischer

**Affiliations:** 1 Department of Psychological and Brain Sciences, Johns Hopkins University, Baltimore, MD USA; 2 Department of Neurology, University of Pennsylvania, Philadelphia, PA USA

**Keywords:** Psychology, Human behaviour

## Abstract

For many odors that we encounter in daily life, we perceive their qualities without being able to specifically identify their sources—an experience termed the “tip-of-the-nose” phenomenon. Does learning an odor’s identity alter our experience of it? Past work has shown that labeling odors can alter how we describe and react to them, but it remains an open question whether such changes extend to the level of perception, making an odor actually *smell different.* Here, in a set of odor classification experiments we tested whether attaching labels to odors can alter their perceptual discriminability. We found that even for odors whose reported similarity changed markedly when their identities were revealed, their discriminability remained unchanged by labels. Our findings indicate that two critical functions of olfaction—parsing the odor environment and supporting the subjective experience of odor qualities—access distinct odor representations within the olfactory processing stream.

## Introduction

Odors are multifaceted stimuli, arising from families of molecules that can vary on thousands of physiochemical dimensions. Odor concentrations in our everyday environments can differ by ten thousand-fold due to air turbulence and distance. Many common odors are made up of hundreds of unique compounds with their own chemical properties that travel through nasal passages at different speeds, interact with receptors with different affinities, and may even interact antagonistically when combined together. Yet, we are able to continually parse the complex olfactory scenes of everyday life, using olfaction to guide how we decide what is safe to eat^1^, how we allocate our visual attention^2,3^, and how we navigate our environment^4,5^.

At the same time, there are key aspects of our olfactory experience that set it apart from the other senses, both in terms of how we access our olfactory knowledge and how we put our olfaction to use in daily life. One striking example is the difficulty that people have in identifying an odor by name^6^, at least in Western societies^7^, even when they can report a host of other qualities about the odor. Whereas it feels trivially easy to recognize that there is a cat nearby when we hear the sound of a meow, or see that an object in the street is a car just by looking, we often struggle to put labels to the smells we experience. Even for a familiar object whose smell seems distinctive—for example, a carrot—when trying to identify it based on smell alone, we might have an experience like: “It’s earthy, clean-smelling, a little sweet… it smells so familiar and I know I’ve smelled it many times in the past, but what is it?!” This experience, referred to as the tip-of-the nose phenomenon, is much more common in olfaction than in other senses. The closest analog in vision might be the experience of trying to recall an acquaintance’s name and feeling the answer insuperably blocked in memory. As in this case, where you might immediately feel that the answer is obvious once you are informed of the person’s name, labeling odors often feels like it snaps the percept into place.

In fact, a long line of work supports the notion that olfactory experience is particularly malleable by non-olfactory information. For example, adding positive or negative labels to odors (e.g., a mixture of isovaleric and butyric acid paired with either the label “parmesan cheese” or “vomit”) induced changes in reported valence so dramatic that the authors proposed labeled odors constituted an olfactory illusion^8,9^. In another study, odors that were paired with either positive or negative labels (e.g., isoamyl acetate with “ripe banana” or “paint thinner”, pyridine with “sea weed” or “rotten fish”, citral with “squeezed lemons” or “insect repellant”) produced different physiological reactions and valence ratings^10^. Labeling odors has similarly been shown to affect hedonic responses to odors as well^11,12^. More recently, work in our own lab has shown that supplying labels for familiar everyday food items can change the perceived relationships among the odors, with some smelling more similar and some smelling less similar after participants learn their identities^13^. Collectively, these studies establish that people’s subjective reports of odor qualities, and the valence of people’s reactions to odors, can be changed by the application of labels. Still, these findings alone are not enough to establish that the labels have actually altered odor perception. Subjective reports are susceptible to influence by higher-order cognition even if perception itself remains unchanged. For example, if someone said “The grass looks greener and the sky looks brighter now that I’ve finished my grant proposal!”, should we be confident that the person did indeed see the grass change hue? Such claims of cognitive penetration in visual perception (i.e., an influence of higher-order knowledge on perceptual experience) have been called into question^14^. Apparent cases of cognitive penetration in vision often arise from the use of measures that fail to isolate perception from experience, and are instead contaminated by biased reports or decision processes^15–18^. Current claims that labels can reshape olfactory experience *at the level of perception* are subject to the same concerns, and it remains an open question whether labels can alter the perception of odors so that they truly smell differently depending on which label is applied.

In the present study, we used an odor mixture discrimination task to determine how well people could differentiate a pair of odors under two different label conditions. Performance on a discrimination task acts as an objective measure of the subjective changes that labels are known to elicit in olfactory perception. Our experiments are based on this underlying premise: if two odors become more perceptually distinct after their labels are revealed, it should become easier to discriminate them from each other. Because the discrimination judgment we employ does not rely on subjective report, and participants are always striving to assign an odor to one of two discrete alternatives, our approach overcomes the issues inherent in subjective reports in order to test whether odor perception itself is reshaped by higher-level knowledge.

## Methods

### Participants

Ninety-four participants took part in the study (63 female, mean age = 19.8 years, all were fluent in English and 66 were native English speakers). All participants were 18–35 years of age and not currently suffering from a stuffy nose (e.g., due to cold or seasonal allergies). Participants confirmed that they had no food allergies. Two participants (one in each experimental condition) were excluded from further analysis after we found that their data were too noisy to estimate a point of subjective equality (PSE), which is necessary for subsequent group analyses (see *Psychometric curve fitting* below). The task lasted approximately one hour. Participants were awarded course credit or $10 cash compensation. All participants provided written informed consent prior to participation. The Johns Hopkins University Institutional Review Board approved all the experimental protocols, and all research was performed in accordance with the relevant guidelines and regulations.

We took two steps to ensure the participants in our study had sufficient olfactory abilities to be included. First, participants were pre-screened for olfactory dysfunction via self-report. Second, we used discrimination performance from the main task below as an indication of whether participants were able to smell the stimuli clearly enough to perform the task. Lapse rates (indicated by performance at the endpoints of the odor mixture continuum) were low, with performance approaching ceiling for pure odors (Fig. 2). These two factors assured us that none of the participants had difficulty smelling the stimuli.

### Odor stimuli

**Figure 1:**
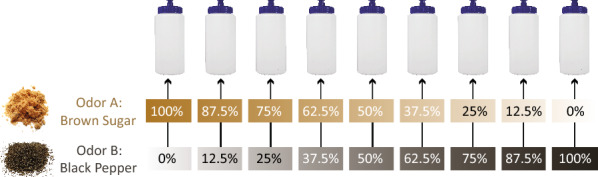
Parametrically varied odor stimuli. We prepared nine opaque bottles containing mixtures of brown sugar (Odor **A**) and black pepper (Odor **B**). Two additional bottles served as standards, one containing only brown sugar and the other containing only black pepper. Standard bottles were labeled **A** and **B**, respectively, and participants were allowed to reference these bottles throughout the experiment to aid in their judgments. The nine unlabeled stimulus bottles contained combinations of brown sugar and black pepper in varying proportions. The weights of the bottles were equated, and the overall quantities of brown sugar and black pepper were chosen to roughly equate the perceived intensity of the two when each was smelled in isolation (see in "Methods" section).

Our stimuli consisted of nine odors delivered in opaque plastic squeeze bottles (Fig. 1). Across the nine bottles, we parametrically varied the relative proportion of two odors—brown sugar and black pepper. We used two readily available commercial products—McCormick Black Peppercorn Grinder and Domino Light Brown Sugar—as our sources for these odorants (see Supplementary Materials). The endpoints of the continuum were pure versions of each (i.e., each bottle contained only brown sugar or only black pepper), and the remaining seven bottles contained incremental mixtures of the two. We selected these two foods as odor sources for a number of reasons. Most importantly, since we intended to test whether the addition of verbal labels alters the discriminability of odors, it was critical to select a pair of odors for which reported similarity changed substantially with the addition of labels. We selected odors that would be rated as somewhat similar when participants did not know their identities, but would be rated as much less similar after labels were applied. To accomplish this selection, we turned to past work in our laboratory that asked participants to rate the similarity of odors both with and without accompanying labels^13^. In that past work, we found that brown sugar and black pepper were among the odor pairs that changed the most with the application of labels, being rated as substantially less similar when participants knew their identities. Several other factors guided our selection as well—we sought odor sources that were shelf-stable, relatively homogeneous, easy to weigh out precisely, and familiar but not highly nameable. The final selection of brown sugar and black pepper took all of these factors into account (Figure S1). Finally, it should be noted that black pepper and brown sugar (like many other real-world odors) may produce small amounts of trigeminal stimulation during sniffing. In the case of black pepper, this is due to the volatile compound piperine naturally present in the plant *piper nigrum* and in the case of brown sugar, it is due to acetic acid produced as a byproduct of the sugar refinement process. Because a trigeminal sensation is produced by both odorants, this alone should not be sufficient to perform the discrimination task without relying on the olfactory perceptual qualities which are the focus of the present study (see Discussion for further considerations regarding trigeminal stimulation).

Ideally, it would have been desirable to measure how labels changed the reported similarity between brown sugar and black pepper in our present sample of participants, to accompany the odor discrimination data in the same groups. However, it was not possible to do so without introducing potential confounds in the mixture classification task detailed below. Because one group in the mixture classification task was given the odor labels prior to the task while the other group was not, any measure of reported similarity of the odors would need to take place at different points in the testing session for the two groups. Given that the central question of this study is whether non-olfactory information influences odor discrimination, it was critical that the experience of the two groups be as similar as possible aside from being informed of the odors’ identities. Thus, we chose not to test the reported similarity of the odors in the same group in which we tested odor discrimination. However, our prior work^13^ offers reassurance that the change in similarity between brown sugar and black pepper with the addition of labels is highly reliable: the effect replicated in four independent sets of participants (independent split halves in each of two separate groups performing within-subjects and between-subjects versions of the experiment).

The quantity of food used to produce the pure odor endpoint (as well as reference standards labeled “A” and “B”) was chosen to make the intensities of the two odors approximately equal (black pepper: 2.3 g; brown sugar: 100 g). Critically though, the mixture of brown sugar and black pepper that smells equally like both (the point of subjective equality; PSE) is inherently subjective and variable across individuals; there is no precise way to infer what an odor’s perceived intensity will be based on its concentration^19^. Thus, there were no perfect quantities of ingredients that we could choose for the pure odors that would balance their intensities for all participants. Rather, we used a procedure of estimating the PSE separately for each participant and accounting for it in order to compute a measure of odor discrimination independent of participants’ varying PSEs (see *Psychometric curve fitting* below). The intermediate odor mixtures were prepared by combining the source foods in ratios that varied from 100% brown sugar / 0% black pepper to 0% brown sugar / 100% black pepper in nine evenly-spaced levels (Fig. 1; expressed as percentages of the quantities of each food used in the two pure endpoints of the continuum). Once mixed, odor stimuli were placed in opaque white squeeze bottles for delivery to participants. Small bags of water were added underneath the foods in each bottle to bring the final weight of the mixture up to ~200 g. This was done so there would be no weight differences among bottles that might affect participants’ judgments while handling the bottles. The reference standards containing the pure brown sugar and black pepper were labeled A and B, respectively. Pepper was ground up in a mortar & pestle. Brown sugar was used as-is. Each item was weighed out in appropriate proportions for each bottle. All mixture bottles were unlabeled and visually indistinguishable. Odors stocks were changed out after every twelve participants.

### Mixture classification task

On each trial of the mixture classification task, participants were presented with one odor mixture bottle to sniff and asked to report which of the two reference odors, Odor A or Odor B, the current odor smelled more like. Participants were allowed to re-smell the reference odors whenever they chose, and there was no time limit placed on their responses. After a participant rated the current odor as more like A or B, they would be given another mixture to rate. All bottles were hidden behind an occluder wall when not in use so that participants could not track bottles across trials. Participants sniffed and evaluated all nine odor mixtures three times each in a randomized order during each block of trials. Each participant completed four blocks of 27 trials each for a total of 108 trials. They were given five minutes of rest in between each block to minimize olfactory fatigue. Participants were reminded to smell the reference odors after each break before resuming the task.

Both the self-pacing of the odor delivery and the breaks interspersed in the data collection session were aimed at mitigating any effects of olfactory fatigue. In their self-paced delivery, participants performed about one trial every 30 s. Our analyses of odor discrimination over the course of the experiment (Fig. 3) confirmed that these measures were successful in avoiding olfactory fatigue, as there was no decrease in discrimination performance over time.

Separate groups of participants completed each version of the mixture classification task (47 participants per condition, with one participant dropped from each condition due to noisy data as detailed below). Participants in the *Unlabeled Odors* condition completed four blocks of the odor mixture classification task without being told the identities of the foods in the bottles. A second group of participants in the *Labeled Odors* condition was informed of the identities of the odors in bottles A and B prior to completing four blocks of the odor mixture classification task.

### Psychometric curve fitting

**Figure 2:**
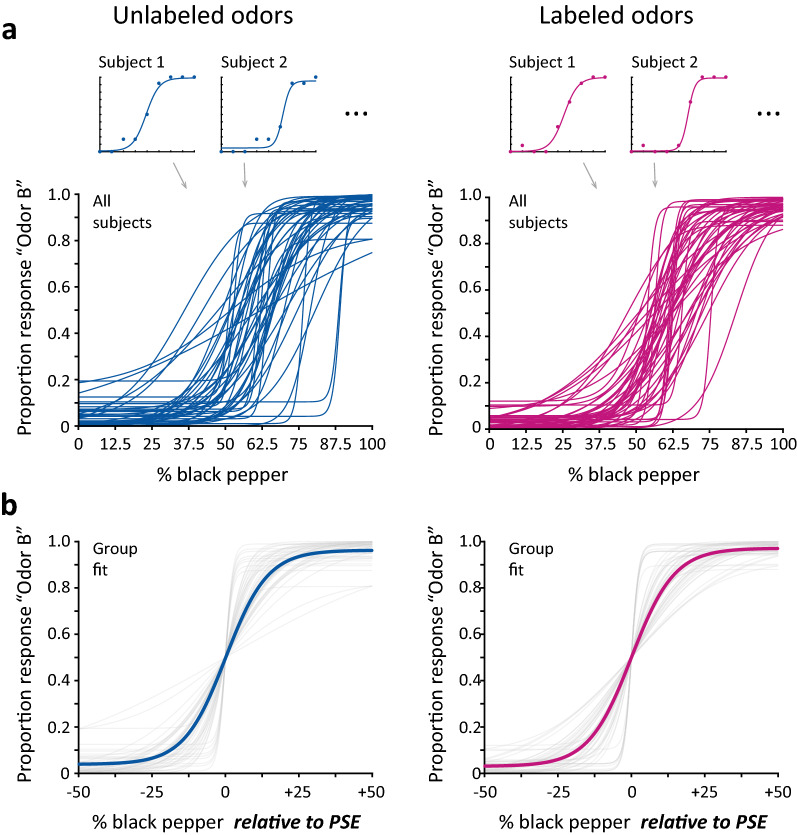
Odor discrimination performance. (**a**) For each individual participant, we plotted the proportion of “B-like” responses as a function of odor mixture, ranging from 0% black pepper / 100% brown sugar to 100% black pepper / 0% brown sugar. We fit a logistic curve to each participant’s individual data, characterizing both the precision of odor discrimination (maximum slope of the curve) and the perceived midpoint of the stimulus range that was equally A-like and B-like (the point of subjective equality; PSE). Data plots from example subjects are shown for both the *Unlabeled Odors* and *Labeled Odors* conditions, and the curve fits from all participants are plotted together to show the variation in PSE. A 2-sample t-test found no difference in the precision of odor discrimination between groups (t(90) = 0.079, *p* = 0.938). (**b**) Group-level psychometric curve fits. After aligning participants’ data by PSE, we fit logistic curves to the aggregated group data for each condition. A permutation test found no difference between groups in the precision of odor discrimination, as given by the maximum slopes of the group-level curve fits (*p* = 0.981).

Participants’ responses from all trials were binned based on the quantity of Odor B (black pepper) that was present in the stimulus for that trial. There were nine bins, ranging from 0% Odor B to 100% Odor B (Fig. 1). We then computed the proportion of trials in each bin on which participants reported the mixture to be more B-like. This procedure was carried out separately for the *Unlabeled Odors* and *Labeled Odors* groups. A logistic curve of the following form was fit to each individual participant’s data:

$$y = \frac{1 - 2c}{{1 + e^{{ - a\left( {x - b} \right)}} }} + c$$

where the *a* parameter scales the slope of the psychometric function, the *b* parameter estimates the point of subjective equality, and the *c* parameter estimates the lapse rate by setting the asymptotes of the curve. This procedure allowed us to independently estimate the point of subjective equality for each participant (Fig. 2a), which was necessary for computing group-level effects. Without adjusting for individual differences in PSE, we might have seen spurious differences in the slopes of the group-level curves that were actually due to averaging participants with varying PSEs. For two participants in our sample (one in each experimental condition), the curve fitting could not establish reliable PSE estimates, and the overall fits of the logistic curves were poor. Because our subsequent analyses critically depended on accounting for individual differences in PSE, we excluded these two participants from further analysis.

To compute the precision of odor discrimination at the group level, we aligned participants’ data to a common space by subtracting each participant’s PSE from the x values for their trials (Fig. 2b). We then fit a group-level logistic curve of the same form as above to characterize odor discrimination for each of the two experimental conditions. We took the slope of the psychometric function (maximum of the derivative) as the measure of discrimination, with higher values (steeper slope) indicating more precise odor discrimination^20^.

### Time course analysis

**Figure 3:**
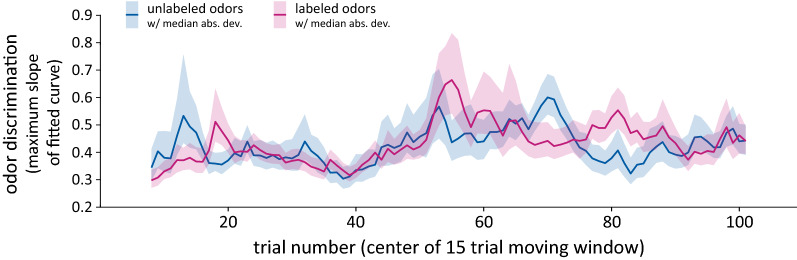
Time-resolved odor discrimination over the course of the experiment. We computed group-level odor discrimination within a fifteen-trial moving window using the PSE-aligned data from each condition. We used a bootstrap resampling procedure to generate confidence intervals around the estimated odor discrimination performance. Solid lines indicate the median of the bootstrapped distribution and the shaded regions show ± 1 median absolute deviation around the median, computed separately in the positive and negative directions. We found a small but significant overall improvement in odor discrimination over time (*p* = 0.019; 1-tailed bootstrap test), but no difference in the rate of improvement between the *Unlabeled Odors* and *Labeled Odors* conditions (*p* = 0.284; 2-tailed bootstrap test).

To evaluate whether odor discrimination performance improved over the course of the experiment, we conducted the same analysis as above, this time within a 15-trial moving window (Fig. 3). Participants’ data were aligned by PSE using estimates from each participant’s full data set. For each window position, which encompassed a time range of 15 trials from within the full set of 108 trials that each participant completed, we collected the trials from all participants and fit a group logistic curve of the same form as above. We took the maximum slope of the fitted curve as a measure of discrimination for the trials within a given window, and by moving the window over the range of 108 trials, we characterized the trend in odor discrimination over the course of the experiment. Note that the first position of the moving window was centered on trial #8 in order to encompass 15 total trials, and the final position of the moving window was centered on trial #101. This analysis was performed separately for each experimental condition.

### Significance testing

We conducted significance testing with non-parametric sampling methods; namely, permutation tests and bootstrap tests^21,22^. To test for a difference in odor discrimination between the two main experimental conditions (Unlabeled Odors and Labeled Odors; Fig. 2b), we generated a permuted null distribution reflecting the difference in slopes between conditions that we would expect to observe by chance, if there was no true difference. Under this null hypothesis, the participants in the two groups arise from the same population, and are exchangeable. Thus, to generate the null distribution, we randomly permuted the group membership of the participants on each of 5,000 iterations, dividing the participants into two groups of equal size on each iteration by random assignment. We then computed the slopes of the group logistic curve fits and recorded the difference between slopes for each iteration. To compute a *p*-value, we found the proportion of the permuted null distribution that was more extreme in absolute value than the true slope difference measured with the original intact group assignments.

To test for a trend in odor discrimination performance over the course of the experiment, we conducted a similar resampling analysis using bootstrapping^21^. On each of 1,000 iterations, we re-ran the time course analysis described above on a random sample of participants drawn with replacement. The sample on each iteration contained the same number of participants as the full set, but could include some participants more than once or some not at all due to the sampling with replacement. This approach used our participants as a model for the underlying population, and the resampling can be thought of as drawing new samples from the population (i.e., running the experiment again). On each iteration, after running the time course analysis we computed the trend over time as the correlation between slope and trial number. The bootstrapping was carried out separately for each of the experimental conditions, and the resulting distributions of correlation values reflected the range of correlations we would expect to observe when running the experiment multiple times.

The time-windowed analysis was prone to occasional poor curve fits due to the limited amount of data within each window and the random sampling of participants. We took two measures to mitigate the impact of poor fits. First, we used the median of the slope values measured at each window position as the measure of its central tendency (displayed as the dark lines in Fig. 3), and we used the median absolute deviation to display the variability (shaded regions in Fig. 3). These measures based on median rather than mean are more robust to extreme outliers^23^. Second, within a given window position, we excluded slopes from iterations in which the computed slope fell more than three median absolute deviations away from the median slope for that window. This approach excluded a small number of iterations in which the curve fits yielded slopes that were extreme outliers, and allowed for a more robust characterization of the trend in odor discrimination over time.

We used bootstrapped samples of the correlation between odor discrimination and trial number to conduct two tests. First, we tested for an overall positive trend over time pooling the two experimental conditions and computing the proportion of bootstrapped correlations that were smaller than zero (1-tailed test, as we were specifically focused on improvement over time). Second, we used bootstrapped samples to test for a difference between conditions, computing the difference in correlation between the two conditions on each iteration. Using the resulting distribution of difference scores, we computed the proportion of the distribution that fell on the opposite side of zero from its mean, multiplied by two (2-tailed test).

## Results

Past work has shown that adding verbal labels to odors can change how people rate, describe, and react to those odors. But do labels alter the most basic levels of odor processing, affecting our ability to distinguish between two odors? To answer this question, we tested whether the addition of labels can change how discriminable two odors are in a two alternative forced choice (2AFC) experiment, avoiding the need to ask participants to explicitly report on the similarity and qualities of the odors. Participants were given two reference odors as a basis for comparison—bottles with 100% brown sugar (labeled “Bottle A”) and 100% black pepper (labeled “Bottle B”)—and completed a series of trials in which they smelled mixtures of A and B in different ratios (Fig. 1). They reported whether each mixture was more A-like or B-like (see in "Methods" section). Two separate groups performed the task, with one group making their judgments without knowing the identities of the reference odors (*Unlabeled Odors* condition), and one group performing the task after being informed of the odors’ identities (*Labeled Odors* condition). Our ultimate goal was to test whether the latter group could more precisely discriminate the two odors by virtue of knowing their identities, in line with the finding that participants *report* the odors as smelling more different after their identities have been supplied^13^.

A challenge in measuring odor discrimination is that the relative intensities of a given pair of odors is subjective and not straightforward to quantify before measuring odor perception on an individual subject basis. In other sensory domains—vision, for example—measurements such as luminance contrast can serve as a basis for comparison across different stimuli. Individual participants still vary in their subjective experiences, but stimulus properties such as luminance contrast can be used as an objective basis for comparing two or more stimuli. In olfaction, on the other hand, detection thresholds and perceived intensity can vary dramatically for different odors presented at the same molecular concentration^19^. In generating our parametrically varied stimulus range, the absolute concentrations of odorants were chosen to approximately equate their perceived intensities at the middle of the stimulus range (see in "Methods" section), but the subjectivity of perceived odor intensity means that participants will vary in what mixture they perceive as the one that is equally A-like and B-like (the point of subjective equality; PSE). It is not meaningful, then, to compute the accuracy of participants’ responses relative to the center of our stimulus distribution. Rather, it was crucial to compute each individual participant’s PSE and account for it, measuring odor discrimination independently of the subjective center of the stimulus range. Accordingly, we began by analyzing individual participants separately. For each of the nine odor mixtures, we computed the proportion of trials in which a participant reported that the mixture was more B-like. When the quantity of black pepper was high (e.g., 87.5%), participants nearly always reported the mixture to smell more B-like, and likewise, when the quantity of pepper was low (e.g., 12.5%), they almost never stated that it was more B-like. Responses for intermediate mixtures captured how sensitive a participant was to incremental changes in the odor mixture.

We fit a psychometric curve to each individual participant’s responses (Fig. 2a; see *Psychometric curve fitting*). The example participants in Fig. 2a are representative of what we generally found—that we could readily fit each participant’s data with a curve that separately captured the precision of odor discrimination (the maximum slope of the curve) and the point of subjective equality (the left–right shift of the curve). For two participants (one in each experimental condition), we could not fit reliable curves to measure the PSE, and we excluded these participants from further analysis (see in "Methods" section). The family of curve fits for each condition in Fig. 2a also confirms the substantial variability in the subjective center of the stimulus range.

To test for a difference in the precision of odor discrimination between conditions, we compared the individual-subject slope estimates between the groups in the two conditions. We found no difference in odor discrimination between the *Unlabeled Odors* and *Labeled Odors* conditions (t(90) = 0.079, *p* = 0.938), indicating that providing participants with the odors’ identities provided no benefit to discriminating between them.

We also performed a group-level analysis of odor discrimination, fitting psychometric functions to the pooled responses from participants in each group after aligning their data by PSE (Fig. 2b; see in "Methods" section). We found nearly identical odor discrimination between the two groups (psychometric slopes of 0.404 for unlabeled odors and 0.402 for labeled odors, where the units are $\frac{{ \Delta \;\text{''}{\text{B - like}}\text{''}\;{\text{response}}}}{{\Delta \;\% \;{\text{black }}\;{\text{pepper}}}}$). A permutation test for a difference in the precision of odor discrimination between groups (see in "Methods" section) found no difference (*p* = 0.981), reinforcing the conclusion that the odors did not become more perceptually discriminable when labeled.

Although the application of labels did not affect overall odor discrimination, labels may have benefitted performance in other ways. In particular, labels could facilitate the process of learning and refining a representation of each reference odor, leading to a greater rate of improvement over time in the classification task. Although we did not observe an overall difference in performance between the *Unlabeled Odors* and *Labeled Odors* conditions, it is possible that a difference emerged late in the testing session and was not detectable in our previous analyses that collapsed across all trials. To evaluate this possibility, we characterized how odor discrimination performance evolved over the course of the experiment. We measured group-level odor discrimination within each condition as described above, this time within a fifteen-trial moving window. Figure 3 shows how odor discrimination performance evolved over the course of the experiment. We conducted two statistical tests to evaluate the trend in performance over time. First, we tested for an overall positive trend in the precision of odor discrimination using a bootstrap resampling procedure (see in "Methods" section), and found that in the aggregated data across the two conditions, there was a subtle but significant improvement over time (*p* = 0.019; 1-tailed bootstrap test). Second, we tested for a difference between the *Unlabeled Odors* and *Labeled Odors* conditions in the rate of improvement. We found no difference (*p* = 0.284; 2-tailed bootstrap test), indicating that the data provide no evidence for a benefit of labels on improving at the discrimination task over time.

Taken together, our results demonstrate with multiple tests that attaching labels to odors does not improve their discriminability. Despite the fact that verbal labels can markedly alter how we interpret and describe odors, they do not modify the odor representations underlying our ability to distinguish two odors from each other.

## Discussion

Our findings show that despite the substantial influence that verbal labels have on the reported qualities of odors, perceptual discriminability of odors remains immune to the influence of labels. These results suggest that odor discrimination and odor evaluation draw on distinct representations in the odor processing hierarchy. In light of our findings, it is intuitively appealing to posit that odor discrimination draws on early stages of odor processing while odor evaluation draws on later stages. When discriminating odors from each other, we may rely on olfactory representations at the stages of the olfactory bulb and/or the anterior piriform cortex where high-fidelity representations of chemical structure could support fine-scaled discrimination among odors. When evaluating the relationships among odors in a more explicit fashion, we might draw on higher-level olfactory representations at the stage of posterior piriform cortex and/or orbitofrontal cortex, where odor representations are enriched with inputs from other sensory modalities and cognitive processes and are subject to being reshaped by verbal labels. This proposal accords with a collection of neuroimaging findings showing that the nature of odor representations evolves from reflecting chemical structure to reflecting subjective qualities over the course of the olfactory processing hierarchy^24–28^. At the same time, other studies have found that orbitofrontal cortex (OFC), typically regarded as a higher-level stage in odor processing, is engaged during odor discrimination judgments^29^, and damage to the OFC impairs odor discrimination but not detection^30^. In light of our findings, future neuroimaging work can use verbal labels as a tool for disentangling odor discrimination and odor evaluation in the brain by tracking how odor representations update with the addition of labels.

Might participants have relied on trigeminal stimulation from a single odorant in the mixtures to perform the task, rather than performing a direct comparison of the relative mixture of the two odorants? Several aspects of the study design and results argue against this possibility. As noted in the Methods, both black pepper and brown sugar produce mild trigeminal activation, meaning that participants could not have simply tracked a single constituent odorant based on its trigeminal activity alone. The pattern of results we observed supports this assessment: if participants were simply tracking trigeminal activation then the endpoints of the odor mixture continuum should be confusable (e.g., 100% brown sugar should be confused with some level of black pepper corresponding to the same level of trigeminal activation). We did not observe such a pattern in our data—instead, discrimination approached perfect performance at both ends of the odor mixture continuum. Additionally, using the single dimension of trigeminal activation as the basis for responses would require participants to establish a threshold (which levels of activation should be reported as more A-like vs. more B-like). Establishing such a threshold would require experience with the range of stimuli in the odor continuum in order to set a midpoint, meaning that performance on early trials should be worse until a stable criterion is set. We did not observe the trend in discrimination performance over early trials in the experiment, further supporting the conclusion that participants relied on a comparison of the proportion of the constituent odors to make their judgments, rather than relying on an intensity judgement of a property (e.g., trigeminal activation) of a single odorant in the mixtures.

A key aspect of the present study is our use of real-world odor stimuli. We made this choice for several key reasons related to the underlying goals of this study. First and foremost, we needed stimuli for which there was a true real-world correspondence between an odor and its label. Many past investigations of odor naming performance have used single-molecule odor stimuli. These odorants *smell like* various familiar objects, but do not reflect the full profile of odorants associated with those objects in our everyday experience. Real-world odors are complex mixtures of tens or hundreds of individual odor molecules that co-occur to produce an identifiable olfactory object. In this study, we prioritized having a true, real-world correspondence between the odor stimuli and the labels we applied, even if it necessitated some tradeoffs in the method of odor delivery during the task.

In sum, our present findings illuminate the level of processing at which higher-order information can reshape olfactory experience: labels change how we rate, describe, and react to odors, but not how we experience the smell of an odor at the most basic level.

## Supplementary Information

Supplementary Information.

## Data Availability

All data presented in this manuscript will be made available in raw form on the laboratory website (www.dynamicperceptionlab.com) upon publication of the paper.
