# Supplementary material for: Odor discrimination is immune to the effects of verbal labels
Source: Sci Rep. 2023 Jan 31;13:1742. doi: 10.1038/s41598-023-28134-w (PMC9889793; doi:10.1038/s41598-023-28134-w)
Supplement: Supplementary file 1 — Supplementary Information. [file 41598_2023_28134_MOESM1_ESM.docx]

Supplementary Materials:

*Information on odor sources:*

To generate the odor mixture stimuli, we used commercial food products widely available at grocery stores in most US cities. For the black pepper odor in our study, we used McCormick Black Pepper Grinder (see *https://www.mccormick.com/spices-and-flavors/herbs-and-spices/grinders/black-peppercorn-grinder* for product details) and for the brown sugar odor we used Domino Light Brown Sugar (see *https://www.dominosugar.com/products/light-brown-sugar* for product details). These products provided odor sources that were both highly consistent and readily available, making it easier for other groups to precisely reproduce the conditions of our experiments.
